# Changes in *var *gene mRNA levels during erythrocytic development in two phenotypically distinct *Plasmodium falciparum *parasites

**DOI:** 10.1186/1475-2875-6-78

**Published:** 2007-06-12

**Authors:** Madeleine Dahlbäck, Thomas Lavstsen, Ali Salanti, Lars Hviid, David E Arnot, Thor G Theander, Morten A Nielsen

**Affiliations:** 1 Centre for Medical Parasitology at Department of International Health, Immunology and Microbiology, University of Copenhagen and Department of Infectious Diseases, Copenhagen University Hospital (Rigshospitalet), Copenhagen, Denmark; 2 Institute of Immunology and Infection Research, School of Biological Sciences, University of Edinburgh, Scotland, UK

## Abstract

**Background:**

The *var *multigene family encodes PfEMP1, which are expressed on the surface of infected erythrocytes and bind to various host endothelial receptors. Antigenic variation of PfEMP1 plays a key role in malaria pathogenesis, a process partially controlled at the level of *var *gene transcription. Transcriptional levels, throughout the intra-erythrocytic cycle, of 59 *var *genes of the NF54 clone were measured simultaneously by quantitative real-time PCR. The timing of *var *transcript abundance, the number of genes transcribed and whether transcripts were correctly spliced for protein expression were determined. Two parasite populations were studied; an unselected population of NF54 and a selected population, NF54VAR2CSA, to compare both the transcription of *var2csa *and the expression pattern of the corresponding protein.

**Methods:**

Synchronized parasites were harvested at different time points along the 48 hours intra-erythrocytic cycle for extraction of RNA and for analysis of expression of variant surface antigens by flow cytometry. Total RNA from each parasite sample was extracted and cDNA synthesized. Quantitative real-time PCR was performed using gene-specific primers for all *var *genes. Samples for flow cytometry were labelled with rabbit IgG targeting DBL5ε of VAR2CSA and serum IgG from malaria-exposed men and pregnant women.

**Results:**

*var *transcripts were detected at all time points of the intra-erythrocytic cycle by quantitative real-time PCR, although transcription peaked in ring-stage parasites. There was no difference in the timing of appearance of group A, B or C transcripts, and dominant and subdominant *var *transcripts appeared to be correctly spliced at all time points. VAR2CSA appeared on the surface of infected erythrocytes 16 hours after invasion, consistent with previous studies of other PfEMP1. Transcription of the pseudogene *var1csa *could not be detected in NF54VAR2CSA cells.

**Conclusion:**

The optimal sampling point for analysis of *var *transcripts using quantitative real-time PCR is the ring-stage, which is encouraging for the analysis of fresh clinical isolates. The data presented here indicate that there is no promiscuous transcription of *var *genes at the individual cell level and that it is possible to correlate dominant transcripts with adhesion phenotype and clinical markers of malaria severity.

## Background

Malaria caused by *Plasmodium falciparum *is a global health problem and the majority of severe clinical cases are found among young children and pregnant women. *Plasmodium falciparum *erythrocyte membrane protein 1 (PfEMP1) is a group of variant surface antigens (VSA) expressed on the surface of infected erythrocytes, thus enabling the parasite to sequester in various endothelial tissues of the host and avoid clearance in the spleen [1,2]. Several receptors have been identified as mediators of the sequestration, e.g. CD36 [3], thrombospondin [4] and ICAM-1, associated with cerebral malaria [5] and chondroitin sulfate A (CSA), the main receptor in the placenta and associated with pregnancy-associated malaria (PAM) [6]. PfEMP1 are encoded by the highly diverse *var *gene family and each parasite genome harbours ~60 of these genes [7-9]. The *var *gene repertoire of the 3D7 parasite [10] can be sub-grouped into three major groups (A, B and C), two intermediate groups (B/A and B/C), and the *var1csa *and *var2csa *sub-families based on promoter regions and chromosomal locations [11,12].

VSA appear to be the main target of antibodies conferring protective immunity against malaria [13-17]. However, antigenic diversity and *var *gene switching have made unambiguous identification of the PfEMP1 molecules that are responsible for specific adhesion phenotypes difficult. A quantitative real-time PCR (Q-RT PCR) method was developed to measure the amount of mRNA being transcribed from 59 different NF54/3D7 *var *genes simultaneously [18]. Using this approach, a particular PfEMP1, VAR2CSA, was identified as the parasite ligand likely to be responsible for CSA binding in the placenta [18,19]. In another study using the same method, the group A *var *genes of 3D7 were associated with severe malaria [20,21]. However, correlating expression of *var *genes to the phenotype of a parasite population can yield equivocal results, and studies on *var *gene transcription have given conflicting results regarding both the number of *var *genes being transcribed and the size of these transcripts. Two reverse transcription (RT)-PCR studies suggested that most or all *var *genes are transcribed in ring-stage parasites, whereas in mature trophozoites all but one *var *gene was silenced. Only the *var *gene actively transcribed in late stages would then be translated into protein to confer the phenotype [22,23]. Other studies have proposed that multiple full-length *var *transcripts are present in ring-stage parasites but not in mature stages, with the exception of the *var1csa *gene which was detectable in both trophozoites and later stages [24,25]. A third group of studies detected multiple full-length transcripts in ring-stage and mature trophozoite parasites in both clonal populations [26] and in single cells [27].

To investigate *var *gene mRNA levels in general, and *var2csa *levels in particular, their abundance and quality were determined in two phenotypically different NF54-derived lines using Q-RT PCR throughout the intra-erythrocytic developmental cycle.

## Methods

### *In vitro *culture

The NF54 clone of *P. falciparum *was cultured as described by Trager and Jensen with modifications [28]. In brief, parasites were maintained in culture using 5% haematocrit of human group O^+ ^blood in parasite medium consisting of RPMI1640 HEPES medium, supplemented with 25 mmol/L sodium bicarbonate (Sigma-Aldrich), 0.125 g/l gentamicin (Invitrogen), and 0.125 g/l Albumax II (Invitrogen). Culture flasks were maintained in a 37°C incubator and gassed with a mixture of 2% oxygen and 5% carbon dioxide in nitrogen. The parasite lines were confirmed to be monoclonal and of same genotype as 3D7, using nested GLURP, and MSP2-specific primers in a single PCR step.

### Selection of VAR2CSA-expressing parasites

The NF54 parasite isolate was repeatedly selected using VAR2CSA-specific antibodies. Briefly, late-stage trophozoites were obtained by gelatin purification and washed twice in RPMI1640. The infected erythrocytes were then incubated for 0.5 hour at 37°C with VAR2CSA-DBL5ε-specific rabbit antiserum and washed three times with medium to remove unbound antibodies. Infected erythrocytes expressing VAR2CSA were isolated by the use of protein G-coupled magnetic beads (Dynal, Invitrogen) on a MACS magnet (Miltenyi Biotec). The suspension of infected erythrocytes and beads was washed three times and added to a culture flask with 4% haematocrit of uninfected erythrocytes in 5 ml culture medium. After 24 hours, the beads were removed by magnetic separation. This was repeated until parasites specifically expressed VAR2CSA on the surface. Expression of the VSA_PAM _phenotype was confirmed by specific binding of parasites to CSA in static binding assays and exclusive recognition of the surface of the infected erythrocytes by antibodies from women exposed to *P. falciparum *during pregnancy. This was done by flow cytometry using a panel of serum samples from *P. falciparum*-exposed men and women and non-exposed Danes as described [29,30].

**Figure 2:**
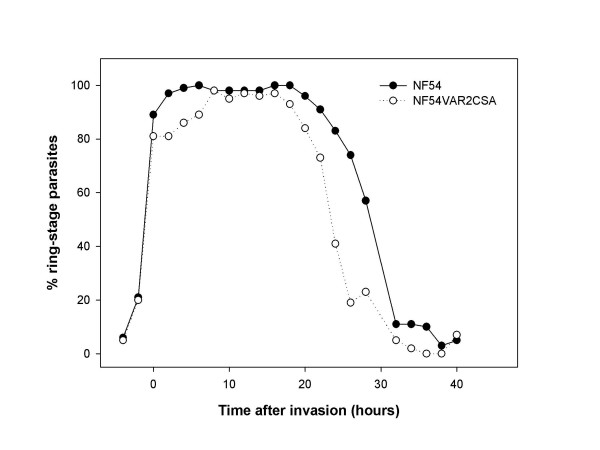
**Percentage of ring-stage parasites in the synchronized populations**. The percentage of ring-stage parasites in the NF54 and NF54VAR2CSA population was determined by Giemsa staining for different time points along the cycle.

NF54 and NF54VAR2CSA cultures were loosely synchronized by gelatine purification of late-stage trophozoites and schizonts. After 48 hours of culture, late-stage trophozoites and schizonts were further enriched on a MACS column to exclude any remaining ring stages, as previously described [31]. This resulted in a synchronous population of parasites with more than 99% late-stage trophozoites and schizonts. Timing of parasite reinvasion was determined by counting the percentage of ring-stage parasites of 300 infected erythrocytes on thin smears stained by Giemsa (Figure 2). For RNA extractions, each parasite line was cultured in 26 different culture flasks at 1% hematocrit (50 μl packed red blood cells) with ~10% parasitaemia after reinvasion. This was done to minimize handling time of the parasite cultures at other time points than during harvesting. The culture was centrifuged and 1 ml of Trizol (Invitrogen) was added to the pellet to preserve RNA. For flow cytometry assays eight different culture flasks were prepared for each isolate. Since separation of ring stages from uninfected erythrocytes is difficult, these cultures were prepared at 0.25% haematocrit with approximately 25% parasitaemia observed after reinvasion to allow for direct staining and flow cytometric analysis.

### Flow cytometry

Flow cytometry was used to assess the timing of VAR2CSA surface expression by each line as described [29,30]. Aliquots of the parasite culture corresponding to 2 × 10^5 ^infected erythrocytes were labelled by ethidium bromide (to allow exclusion of remaining uninfected erythrocytes). Pools of human sera and rabbit VAR2CSA-specific serum were used for IgG surface-staining of infected erythrocytes. Infected erythrocytes were sequentially exposed to 5 μl pooled human serum, and 1 μl goat anti-human IgG (Vector) in a total volume of 100 μl. For surface staining by rabbit VAR2CSA DBL5ε -specific IgG, samples were sequentially exposed to 20 μl rabbit serum pre-absorbed against uninfected erythrocytes, 1 μl biotinylated sheep anti-rabbit IgG (The Binding Site) and 0.5 μl streptavidin-FITC (BD Pharmingen). All incubations were performed in a total volume of 100 μl PBS with 2% FCS for 30 min. Samples were washed twice with 3 ml PBS with 2% FCS in between each incubation. Data from a minimum of 5,000 infected erythrocytes were acquired using a FACScan instrument (BD Biosciences). A batch of uninfected erythrocytes was analysed to exclude IgG binding to erythrocyte antigens. All samples relating to a particular time point were stained and processed immediately after removal from the 37°C incubator.

### RNA purification and cDNA synthesis

Synchronized infected erythrocytes were harvested every 2 hours throughout the intra-erythrocytic life cycle for extraction of total RNA and cDNA synthesis as described elsewhere [18]. A representative subset of samples that described the profile of the transcript abundances was further analysed by Q-RT PCR. In brief, 50 μl of packed erythrocytes with ~10% parasitaemia was dissolved in 1 ml of Trizol (Invitrogen) and RNA was prepared according to the manufacturer's instruction. RNA pellets were dissolved in 10 μl of RNase-free water and treated with *DNase*I (Sigma-Aldrich) for 25 min at room temperature, followed by 10 min heat inactivation at 65°C. All RNA samples were subsequently tested in real-time PCR for contamination with genomic DNA using a primer set for the *seryl-tRNA synthetase *gene (Table 1). DNA-free RNA samples were used for synthesis of cDNA by reverse transcriptase (Superscript II, Invitrogen) and random hexamer primers as described by the manufacturer. The cDNA content was confirmed by real-time PCR using primers for the *seryl-tRNA synthetase *gene.

**Table 1:** Specific gene primers used for Q-RT PCR. Primers used to specifically amplify *var *genes, a pseudogene and control genes in Q-RT PCR, in addition to primer sets designed by Salanti *et al*. [18]. Primers from Salanti *et al*. that were used in this study (but not shown in table): no. 4–12, 15, 17–23, 25–28, 30, 34–39, 41, 43, 45, 46, 49–55, 91–96, and 97.

**Primer set**	**Forward primer**	**Reverse primer**	**Target gene**
1 new	TAAATTGCTCAATCGCGAAG	CATCGTCATCATCGTCATCA	PFA0005w
16	AAAGCCACTAGCGAGGGTAA	TGTTTTTGCCCACTCCTGTA	PFL1955w
24	AGGACAACACGGATGAGACA	AGCAGTGTTGTCGCCATTAG	PFC0005w
32	ACCTAATGGCGAAAACCAAG	ACACTTGCCTTCATCCACTG	PFD1000c
44 new	CCTACACTCACCTCCCCCTA	ACACTCACACGCCTCATCAT	PFF1580c
58 new	ACAACAATTTCGCAAGCAAG	TTCCTCTGCCTCCTCTTCAT	PFI1830c
66	CCTAAAAAGGACGCAGAAGG	CCAGCAACACTACCACCAGT	PF08_0107
67	AAGGGAAGATTGGTGGACAG	AGGGGGATCAGTATCACGTC	PFF0020c
90^a^	TCAATTTGATAAAGTGGAACAATTC	GCGTTGTTTAAAGCTCCTGA	PF07_0073
98	ATGGTGGCAAACTTGTGAGA	TCCAATTGGTCTCCTTGACA	PF08_0140
99	AGGGAGCATCAGGTGGTAGT	GCTGTGCATGCTTTTTCATT	MAL7P1.50
100	GATCAAAAGAGGCGGAGAAG	TTCCAATTGGGGAATTTTTC	PFI0005w
101	CAAGAGACACAACCGGAAGA	CACTTCCAATTGGGGAATTT	PFA0765c
498/499	AATCAGAAAAGTGTAATTGCAGGAG	TTTACTATCATCACTGACACGCATT	MAL7P1.212
500/501	GTCCTCTATGTGGAGTGAAAAAGAA	AGTACCGTTATCTGGGTTTATAGGC	MAL8P1.220
392/393^b^	AAACGAAAGCGAAGCAGAAT	CACCTTCACTCCTTGCCATA	PFF0005c
334/335	AGCCCAATCGGAAGGTAAGT	TTCATAGCTTCTAGCGCCTT	PFL0030c
UTR var2csa	CACGACATTAACAATACATGCAGA	CATTGCATTCACAGACATTGG	PFL0030c
390/391^c^	AATAATACCAGTGACATTCTGCAAAA	ACACGTAAAAGGTCCACAGGTG	PFL0030c
535/536^c^	CCAGCAGGACCAGCAACAGATAGTGGC	CCACTTCTATATGGTATGTACCTATTTTTG	PFL0935c
496/497^c^	AATCACTTCCGCAACCCCCACG	CTGAAAAGGTCCACACGAGG	PFD0615c
MSP1	ACAACTGAAGATGGGGGTCA	TTTTGGTGGTGATGGTTGTG	PFI1475w

a. Endogeneous control gene, *seryl-tRNA synthetase*.

b. Pseudogene.

c. Primers designed to target flanking regions of the intron (cross-intron primers).

### Primer design

Transcription of all the *var *genes was determined using primers originally published by Salanti *et al*. [18], although some have since been redesigned (Table 1). Table 1 also contains primers designed to target new *var *genes identified in the *P. falciparum *genome database [32] and primers for additional genes used as controls (*msp1 *– PFI1475w and *seryl-tRNA synthetase *– PF07_0073). All primers were initially tested on genomic DNA to determine the 'amplification efficiency' compared to the amplification of the internal control, *seryl-tRNA synthetase*. Primer pairs that varied more than two Ct values from the Ct value of the internal control were redesigned. Primers for detecting spliced *var *transcripts were designed to target the flanking regions of the intron (cross-intron primers). The 'amplification efficiency' of these cross-intron primers was not determined. However, all cross-intron primers were tested on genomic DNA in conventional PCR with increased elongation time and the PCR products were fractionated by agarose gel electrophoresis. The cross-intron products amplified from cDNA by Q-RT PCR were also fractionated by agarose gel electrophoresis.

### Quantitative real-time PCR

All Q-RT PCR measurements were performed using the Rotorgene RG-3000 system (Corbett Research). Reactions were prepared in volumes of 20 μl using QuantiTect SYBR Green PCR master mix (QIAGEN) and a primer concentration of 1 μM. The following PCR cycling conditions were used: initial heat activation step at 95°C for 15 min, followed by 40 cycles of 95°C for 30 s, 55°C for 30 s and 68°C for 40 s with a final extension at 68°C for 40 s. The amplification specificity for each primer pair was determined by melting-curve analysis of each PCR product.

### Quantitative analysis

Gene specific standard curves were generated by determining the 'amplification efficiency' relative to the single copy endogeneous control gene (*seryl-tRNA synthetase*), based on real-time measurements of 10-fold dilutions of genomic DNA. Transcript abundance was determined according to the ΔCt (cycle threshold) method, in which the Ct value for each specific gene was compared with that of the endogeneous control, and by calculating the relative copy number, based on the specific standard curves for each gene. Standard curves for the cross-intron primers were not generated.

## Results

### Transcripts of *var *genes were detected throughout the intra-erythrocytic life cycle

**Figure 1:**
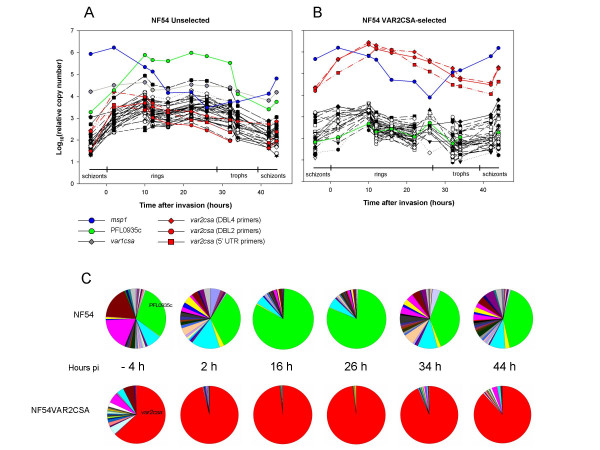
**Transcriptional profiles of NF54/3D7 *var *genes in two phenotypically different parasites**. Quantification of all the *var *transcripts normalized against *seryl-tRNA synthetase *and shown as log10(relative copy number) in (A) and (B) and as pie charts in (C) at different time points during the intra-erythrocytic life cycle of NF54 and NF54VAR2CSA. (C) shows only expression of full-length *var *genes. (A) and (B):blue – *msp1 *(was used for monitoring the different stages), green – PFL0935c (dominant transcript in NF54), grey – *var1csa*, red diamonds – *var2csa *(DBL4 primers), red circles – *var2csa *(DBL2 primers), red rectangles – *var2csa *(5' UTR primers). (C): light green – PFL0935c, red – *var2csa *(DBL2 primers). pi – post invasion

Using Q-RT PCR the *var *transcriptome pattern throughout the intra-erythrocytic life cycle was investigated for two parasite cultures, NF54 and NF54VAR2CSA. Figure 1A and 1B show the transcript abundance of 59 full-length *var *genes and two *var *pseudogenes in NF54/3D7 as relative copy numbers normalized to the internal control, *seryl-tRNA synthetase *(PF07_0073). The percentage of ring-stage parasites was determined by Giemsa staining (Figure 2), in addition to measurements of *msp1 *(PFI1475w) transcript abundance to control for stage specificity of each parasite population (Figure 1A and 1B). For both cultures there was a clear peak in transcript abundance at ring stage, ~5–25 hours after invasion. Figure 1C shows the transcript abundance of each full-length *var *gene relative to total number of *var *transcripts, in which the proportion of the most abundant transcript was higher at ring stage compared to the schizont stage.

The most abundant *var *transcript in NF54 ring-stage parasites was PFL0935c (Figure 1A and 1C), which in the 3D7 genome is a centromeric *var *gene on chromosome 12 with a group B 5' UTR promoter according to the grouping of 3D7 *var *genes [10-12]. Two additional *var *genes were transcribed at high levels in NF54, PFL1960w (group C, localized on chromosome 12 in the 3D7 genome) and PF08_0106 (group B/C, on chromosome 8). By contrast, NF54VAR2CSA represents a highly homogeneous population, which transcribes almost solely *var2csa *(PFL0030c, on chromosome 12). All other *var *genes were transcribed at low levels or below the detection level (Figure 1B). Three primer sets targeting DBL2, DBL4, and the 5' UTR of *var2csa*, were used and the results obtained with these primers were tightly correlated (Figure 1B). Transcripts of *var2csa *were also detected in the unselected NF54 at low levels, similar to those of the less abundant *var *mRNA

**Figure 3:**
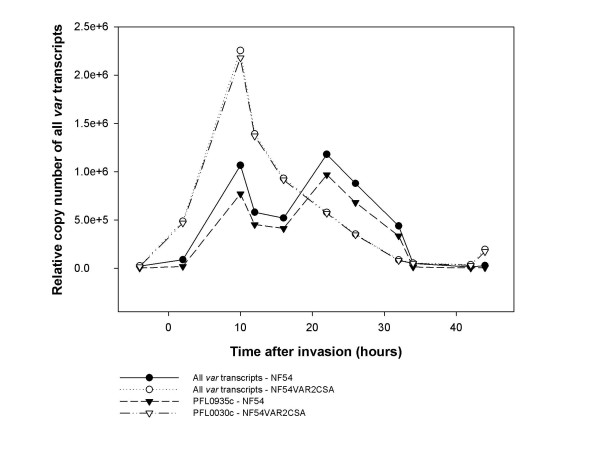
**Total number of *var *transcripts in NF54 and NF54VAR2CSA**. Total *var *transcription is shown as relative copy number for both NF54 and NF54VAR2CSA. Total *var *transcription of both populations corresponds to the expression of the most abundant transcript, PFL0935c and *var2csa *(PFL0030c), respectively. The most striking difference in transcription is the slightly earlier distinctive peak for *var2csa *(PFL0030c) in NF54VAR2CSA compared to the broader wave-like appearance of *var *transcription in NF54. All measurements at the different time points were normalized against *seryl-tRNA synthetase*.

The transcriptional time profile of *var2csa *appeared slightly different from the other *var *genes. This is most evident in Figure 3 showing relative copy numbers of the most abundant *var *transcript as well as total *var *transcripts in both populations. The transcript abundance peak of *var2csa *in both parasite populations emerges slightly earlier in the ring stage of the parasite development and also declines faster than for the other *var *genes (Figure 1A and 3). In addition, the broad transcript abundance peak in NF54 seems to consist of two waves, a pattern which was observed for all the *var *genes (Figure 1A and 3). No differences between the various *var *gene groups (A, B and C) were detected regarding appearance and disappearance of transcripts.

The transcript abundance of two truncated sub-telomeric *var *pseudogenes of NF54/3D7, *var1csa *(PFE1640w, on chromosome 5, lacks intron and exon II) and PFF0005c (group A pseudogene, on chromosome 6, truncated in DBL1α), was also determined. *var1csa*, which has been found to be constitutively transcribed in many parasites irrespective of binding phenotype [25], was constitutively transcribed at high levels in NF54 but barely detected in NF54VAR2CSA (Figure 1A and 1B, respectively). The more recently discovered *var *pseudogene, PFF0005c, was detected at a few time points at very low levels in both isolates.

### Dominant and subdominant *var *genes are transcribed as spliced mRNA throughout the intra-erythrocytic life cycle

**Figure 4:**
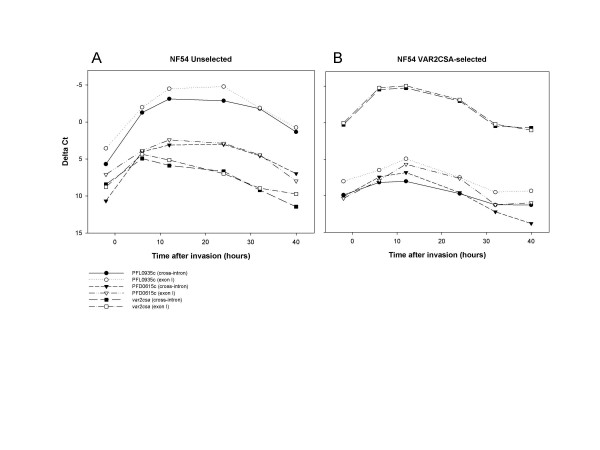
**Both dominant and subdominant *var *genes seem to be correctly spliced throughout the whole intra-erythrocytic life cycle**. Transcript abundance measured by cross-intron primers and exon I primers for the dominant and two subdominant *var *genes are shown as ΔCt values for (A) NF54 and (B) NF54VAR2CSA.

To investigate whether *var *transcripts are correctly spliced throughout the intra-erythrocytic life cycle, specific cross-intron primers were designed (Table 1) for the dominant (most abundant) gene in each isolate (PFL0935c and *var2csa*) and a variant that was subdominant (less abundant) in both parasite populations (PFD0615c, group C, on chromosome 4). The transcript abundance profiles measured by the cross-intron primers targeting the dominant and subdominant genes were similar to the profiles given by the corresponding exon I primers. This indicates both that the two sets of primers were recognizing the same transcript and that most, if not all, detectable transcripts were already spliced (Figure 4). The difference in ΔCt value between the primer sets for PFL0935c (Figure 4A) was most likely due to differential primer bias.

### Timing of VAR2CSA expression on the surface of NF54VAR2CSA

**Figure 5:**
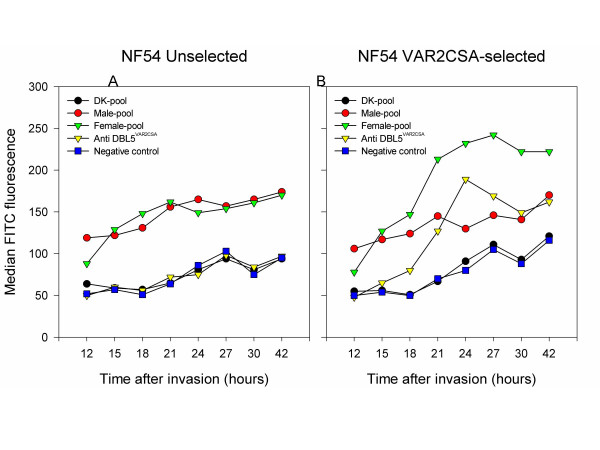
**Timing of VAR2CSA surface expression**. Surface staining of infected erythrocytes selected with VAR2CSA-specific rabbit antibodies, NF54VAR2CSA, and the unselected control, NF54. The median of FITC fluorescence as measured by flow cytometry is shown for each serum sample. Pools of human sera from *P. falciparum *exposed females and males, and unexposed Danes (DK-pool) were used as primary antibodies. VAR2CSA expression was confirmed by staining with VAR2CSA DBL5ε-specific rabbit antibodies and irrelevant rabbit immune serum was used as negative control. Samples were processed and run simultaneously at individual time points.

Parasites from infected placentae and CSA-selected late-stage parasites are recognized by human immune sera in a sex-specific manner. This was also the case for NF54VAR2CSA (Figure 5B). As expected, there was no difference in the recognition of unselected NF54 by female and male sera (Figure 5A). The flow cytometry data confirmed published findings that VAR2CSA, like other PfEMP1, appears on the surface of infected erythrocytes several hours after the first detection of *var *transcription (Figure 1B and 5B). Surface-exposed VAR2CSA was detected by VAR2CSA-specific rabbit antibodies and female serum from a malaria endemic area about 18 hours after invasion, and the amount of PfEMP1 reached a plateau which seemed to remain constant throughout the later stages. Male immune sera, on the other hand, showed the same lack of recognition of NF54VAR2CSA as was seen in unselected NF54.

## Discussion

Transcript levels of *var *genes were analyzed throughout asexual parasite development using an improved version of the Q-RT PCR method originally published by Salanti *et al*. [18]. Primer pair coverage was extended to optimize quantitative measurement of mRNA transcribed from 59 full-length *var *genes and two pseudogenes in the NF54/3D7 genome. Two phenotypically distinct NF54-derived lines, NF54 (unselected) and NF54VAR2CSA (selected for VAR2CSA expression) showed abundance peaks of dominant and subdominant *var *transcripts at ring stage, although the relative abundance of the dominant transcript in each population was higher in ring stage and early trophozoites than in schizonts. This can be explained by the increased uncertainty of measurement when transcript abundance is very low.

Unselected NF54 parasites are clearly a mixture of subpopulations expressing different *var *genes (Figure 1). However, after repeated selection, the *var2csa *gene has become the overwhelmingly dominant transcript and constitutes >98% of all detected *var *transcripts. This pattern of exclusive expression of the *var2csa *gene (Figure 1C) supports the general model of mutually exclusive transcription of *var *genes. Such a general model was proposed after experiments showed that exclusive transcription of activated group B and C 5' UTR promoters occurred following transfection [33,34]. It is likely that a significant proportion of the unselected ring-stage NF54 is exclusively expressing the dominant group B *var *transcript. However, this is not as apparent in this line as in the NF54VAR2CSA due to the heterogeneity of this unselected population. While it remains to be proven that *var *transcription in circulating parasites in the bloodstream can always be correlated to the binding phenotype of the corresponding trophozoite population, this study supports such a conclusion. Another recent study has shown that the genotypes of the circulating ring-stage parasites represent the types of the mature parasites sequestering in most tissues [35].

Improved quantification of transcript abundances of each *var *gene obtained by Q-RT PCR and use of cross-intron primers also support a revision of the view that *var *transcripts are exclusively restricted to the ring stage and that there is promiscuous transcription of many *var *genes in ring-stage parasites [24,36]. New data, reported here and in other recent publications [37], indicate that the so-called 'loose' transcription of several *var *transcripts per infected erythrocyte results from cross-hybridization artefacts and the background of gene expression from minority populations in cultures which are not expressing the dominant, selected *var *gene.

Truncated or unspliced transcripts in parasites have also been reported [8,38]. In this study, cross-intron primers for three different *var *genes, *var2csa *(PFL0030c), a group B gene (PFL0935c) and a group C gene (PFD0615c) all showed splicing of exon I to exon II in both the unselected and VAR2CSA-selected NF54 parasites throughout the whole cycle, indicating that these *var *genes are normally expressed as correctly spliced transcripts (Figure 4). The fact that the transcription levels of the four different *var2csa*-specific primers were tightly correlated further indicates that the transcripts are not only correctly spliced but also probably full-length at all stages. This is contrary to a conclusion drawn in an earlier study in which a difference between the levels of transcription detected by cross-intron primers and exon I primers of some *var *genes using Q-RT PCR were reported [38]. This discrepancy could be due to the 'amplification bias' of the cross-intron primers, and the difficulties of designing unique primers targeting the exon II. However it is worth noting that promiscuous transcription in any form is incompatible with transfection experiments showing the exclusive expression of a single *var *gene regulated at a pre-transcriptional level [33,34,39,40].

It has also been suggested that there is a differential repression of the various *var *gene groups based on their promoters [41]. The group B genes (subtelomeric) were shown to be transcribed 0–10 hours after invasion, and turned off when the parasites reached later stages, whereas the group C (chromosome-central) transcripts were still detected 4–8 hours later than the subtelomeric genes. This differential transcription pattern was not seen here. All *var *genes within group A, B or C in the unselected NF54 population showed the same abundance profile regardless of promoter.

Both *var1csa *and *var2csa *have unique promoters (group D and E respectively, [11,12], and both these genes showed distinctive expression profiles. *var1csa *transcription remains anomalous and poorly understood. *var1csa *was constitutively transcribed in the unselected NF54 line, a result reported previously for several *P. falciparum *isolates, regardless of their receptor binding phenotype [25,38,42]. However, *var1csa *transcription was not detected in any stages in NF54VAR2CSA. This observation is most simply explained by a *var1csa*-expressing subpopulation of NF54 having been removed by VAR2CSA selection. This interpretation further entails that only one *var *gene is transcribed per infected erythrocyte and the *var1csa *gene is restrained from transcription by the silencing mechanisms operating on all *var *genes that is imposed when another *var *gene, in this case *var2csa*, is expressed.

The essentially exclusive expression of a single *var *gene that is observed in the NF54VAR2CSA parasites, where *var2csa *constitutes 98% of total *var *gene abundance, has previously only been observed in *P. falciparum *cultures transfected with drug-selectable marker genes [33,34]. In these experiments, transgenic parasite lines use a *var *promoter to drive expression of a drug resistance gene rather than a PfEMP1 protein. Drug selection followed by cloning and analysis of resistant lines indicates that the transfected recombinant promoter is activated in these lines and that all other *var *promoters are silenced. There is disagreement as to whether a single active *var *promoter is sufficient to silence all other promoters [34] or whether the system of allelic exclusion requires both the 5' *var *promoter and a promoter located downstream in the *var *gene intron to interact in some way to silence transcription [43,44].

Transcription of *var1csa *in the unselected NF54 presumably results from a subpopulation of parasites in which other *var *genes are silenced. The relatively constitutive transcription of *var1csa *may result from a dysfunction of the transcriptional regulation related to the cell cycle. As *var1csa *has an upstream promoter but is truncated at exon I, it can be hypothesized that its 5' UTR promoter, as previously demonstrated [34] is needed for exclusive transcription of a single *var *gene whereas the intron/exon II/3' UTR is needed for the observed wave shaped transcriptional regulation throughout the cell cycle. Yet, the abundance profile of *var1csa *during the complete intra-erythrocytic cycle in other isolates than 3D7 and NF54 remains unclear, and more studies are needed to elucidate the role of this *var *gene. Furthermore, it is not known whether the apparent lack of other pseudogene-transcribing subpopulations, here represented by PFF0005c, can be related to the difference in the 5' UTR promoter.

*var2csa *had a pattern of changes in mRNA abundance similar to that of the other *var *genes being transcribed in the unselected NF54 line. However, the abundance profile indicated that its transcription peaks earlier in the ring stage than the other *var *genes. Since the timing of the cycle of both lines were tightly correlated according to the Giemsa stain assessment, this observation is probably not due to major differences in synchronization. The protein expression of VAR2CSA did not seem to diverge from earlier reports showing that PfEMP1 molecules appear to be present on the infected erythrocyte membrane around 16–18 hours after invasion [45-47]. As expected, the recognition pattern of NF54VAR2CSA by rabbit antibodies raised against recombinant-produced DBL5ε of VAR2CSA was correlated with the recognition of the immune sera of malaria-exposed multi-gravidae women from a malaria endemic area.

## Conclusion

*var *transcription can be measured by Q-RT PCR at all stages throughout the intra-erythrocytic life cycle. However, to identify the dominant transcript in a parasite population it is critical to perform the analysis on ring-stage parasites when transcript levels peak. The findings that a highly homogeneous parasite population transcribes essentially one *var *gene at all time points and that the *var2csa *gene, a group B *var *gene and a group C *var *gene are all expressed as correctly spliced transcripts at all intra-erythrocytic stages suggest that previous reports of promiscuous transcription of many *var *genes in a single parasite might represent experimental artefacts resulting from the presence of heterogeneous parasite populations.

## Authors' contributions

MD carried out the RNA extractions, cDNA synthesis and all the Q-RT PCR experiments, analysed the data and wrote the manuscript. TL participated in the design of the study, helped with primer design, the Q-RT PCR and to draft the manuscript. AS contributed to the study design and helped to draft the manuscript. LH, DEA and TGT helped to finalise the manuscript. MAN participated in the study design, carried out all the parasite work and flow cytometry experiments and helped to finalise the manuscript. All authors read and approved the final manuscript.
